# Signal Quality Index Based on Template Cross-Correlation in Multimodal Biosignal Chair for Smart Healthcare

**DOI:** 10.3390/s21227564

**Published:** 2021-11-14

**Authors:** Seunghyeok Hong, Jeong Heo, Kwang Suk Park

**Affiliations:** 1 Division of Data Science, The University of Suwon, Wauan-gil 17, Hwaseong-si 18562, Korea; shongdr@gmail.com; 2 LG Electronics CTO Division Future Technology Center A.I. Lab., Seoul 06763, Korea; jeong.heo@lge.com; 3 Department of Biomedical Engineering, College of Medicine, Seoul National University, Seoul 03080, Korea

**Keywords:** comfortable measurement, physiological measurement, biological SQI, capacitive ECG, unobtrusive PPG, BCG

## Abstract

We investigated the effects of a quality screening method on unconstrained measured signals, including electrocardiogram (ECG), photoplethysmogram (PPG), and ballistocardiogram (BCG) signals, in our collective chair system for smart healthcare. Such an investigation is necessary because unattached or unbound sensors have weaker connections to body parts than do conventional methods. Using the biosignal chair, the physiological signals collected during sessions included a virtual driving task, a physically powered wheelchair drive, and three types of body motions. The signal quality index was defined by the similarity between the observed signals and noise-free signals from the perspective of the cross-correlations of coefficients with appropriate individual templates. The goal of the index was to qualify signals without a reference signal to assess the practical use of the chair in daily life. As expected, motion artifacts have adverse effects on the stability of physiological signals. However, we were able to observe a supplementary relationship between sensors depending on each movement trait. Except for extreme movements, the signal quality and estimated heart rate (HR) remained within the range of criteria usable for status monitoring. By investigating the signal reliability, we were able to confirm the suitability of using the unconstrained biosignal chair to collect real-life measurements to improve safety and healthcare.

## 1. Introduction

Every time a person sits on a seat of a vehicle or a powered chair, that person could be monitored through multiple vital signs. This is the goal of our research into developing a signal quality index and designing a chair that provides biomedical signal measurements—a biosignal chair.

People with industrialized lifestyles sit in chairs frequently; thus, a chair that could provide biosignals could not only confirm vital signs but also acquire a large amount of cumulative daily physiological information including the data in a self-driving car.

Accumulating daily data outside of a hospital environment increases the potential to reveal solutions for preventing drowsy driving [1] or diseases [2] such as cardiovascular disease (e.g., heart attacks and stroke), cancer, diabetes, epilepsy, seizures, and obesity. Despite their importance, regular biosignal data such as an electrocardiogram (ECG), photoplethysmogram (PPG), and ballistocardiogram (BCG) signals are rarely recorded during people’s daily lives because long-term biosignal measurements have fixed procedures that require the cooperation of motivated users.

Therefore, fixed-on-environment systems (e.g., chairs and beds) and wearable sensors have been developed to minimize the requirement for cooperation. Measurements using sensors installed in furniture have an important advantage: they do not require users to wear sensors. Among furniture items, a chair system can be utilized not only at home but at work or in vehicles (e.g., cars and airplanes). Beyond health monitoring, conditions such as drowsiness while driving a vehicle could also be detected by chair systems [3] to help prevent accidents. The continuity of gathered physiological information is one of the major motivations of chair system development.

Additionally, the capacity to measure multiple physiological signals at multiple sites is another advantage of fixed-on-environment systems compared to wearable sensors, which have weight and space limitations. The ability to use more sensors increases the probability of recording reliable signals given the various situations that occur during the measurements. Various modalities could provide extended information due to the relationships between different signals.

Previous chair-based studies have suggested using combinations of the following physiological signals: capacitively coupled electrocardiogram (cECG) [4,5], clothes-transmittable photoplethysmogram (cPPG) [6], and ballistocardiogram (BCG) [7]. The experiments have demonstrated the usefulness of chair systems in estimating heart rate (HR) and blood pressure [8]. The main advantage in simultaneously measuring PPG and BCG with ECG is that the multiple signals increase the opportunity for measuring uncorrupted data by selecting the optimal channel among the multiple modalities for HR estimations. Additionally, the derivation of pulse arrival time or pulse transit time has become a promising method for estimating blood pressure because it frees users from the inconvenience of cuff-based measurement methods.

In previous studies of unconstrained chair-based measurements, cECG signals were collected using electroconductive sensors on the back of the seat even after conduction through external interface materials, including clothes [9]. After the electrical signal for cardiac muscle contractions for the left ventricle of a heart, the force in reaction to blood pumped from the heart into the vessels has been measured using physical sensors (e.g., polyvinylidene fluoride (PVDF) [10], electromechanical film (EMFi) [11] sensors, load cells [12], and accelerometers [13]) in BCG applications involving furniture while resting. Concurrently, blood changes can be measured by unobtrusive cPPG [6] by light transmission through clothes, tissues, and vessels at various body parts (thigh and back) that contact a chair. The major limitation of cPPG is the measurability when faced with different types of clothes because the measurement depends on light permeability. 

A contributing factor in this limitation is the difficulty of increasing the intensity of light-emitting diodes (LED) and the sensitivity of photodetectors (PD). In a previous cPPG chair study, the researchers revealed a potential safety issue: despite additional heat sink configurations, LED temperatures rise with the increasing current needed to penetrate thick clothes [7]. We believe we have solved the heat problem in a cPPG system for practical use.

We developed a chair system that unobtrusively measures cECG, BCG, and cPPG after solving the weaknesses of the former system. During preliminary experiments, various motions highly affect sensor signals without fixations. Furthermore, by utilizing the mobility of motorized wheelchairs, we were able to investigate the relationship between various movement velocities and the quality of each signal. In this dynamic situation, we observed different signal changes in electrocardiogram and cPPG signals based on various contact methods.

The main contributions of this study are as follows:

- We developed a general and intuitional signal quality index for biosignals measured by unobtrusive systems.
- A solution for the heat yielded by high-power LEDs in successful clothes-permittable PPG with an LED-controlling system.
- Arm movement, real driving, and long-term driving experiments with the zero-effort measurements of biosignals through a chair.
- We observed the feasibility of cPPG sensors to supplement the weakness of cECG and BCG sensors for a specific motion.
- Performance of HR estimation is evaluated using a screening method by signal quality index.
- HR diagnostic efficacy of the proposed approaches is compared with that of conventional methods, and it shows a better performance.

The remaining sections of this paper are organized as follows: In Section 2, we describe the materials used for the biosignal measurement chair. In Section 3, we conducted two experiments, one involving various body motions and one involving powered wheelchair movements. In Section 4, we describe our findings and discuss the screening effects of the signal quality index. Section 5 concludes the paper.

## 2. Materials

### 2.1. System Structure

**Figure 1:**
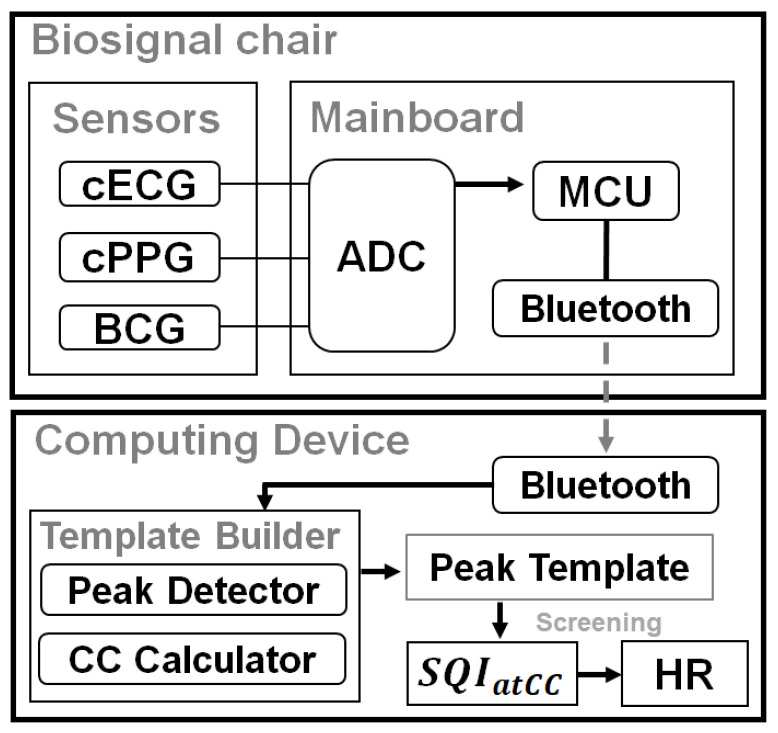
System structure and process. ADC: analog-to-digital converter, MCU: microcontroller unit, *atCC*: averaged template cross-correlation, SQI: signal quality index, HR: heart rate.

**Figure 2:**
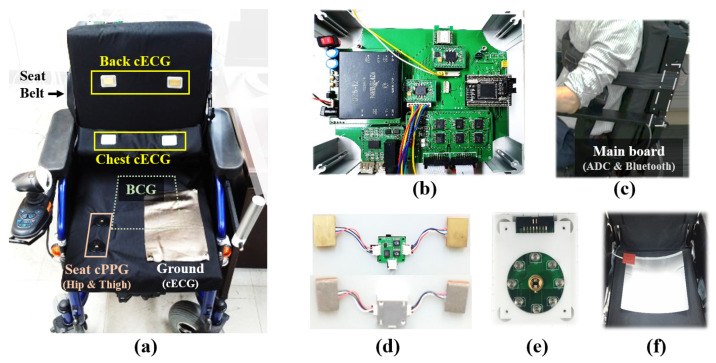
Biosignal chair. (**a**) Sensor locations. (**b**) Mainboard. (**c**) Side view of the wheelchair fastening seatbelt. (**d**) cECG electrodes with the casing used for the signal enhancement circuit. (**e**) cPPG probe with 8 LEDs and a PD. (**f**) EMFi sensor below the seat base for BCG detection.

The chair sensor part for zero-effort measurements of biomedical information was composed of two cECG probes, two cPPG probes, and an EMFi sensor for BCG (Figure 1). The main printed circuit board (Figure 2c) includes a microcontroller unit (MCU) ATMEGA 128 (ATMEL Co., SAN Jose, CA, USA), two eight-channel analog-to-digital converters (AD7606-8, Analog Devices, Wilmington, MA, USA), and a Bluetooth module (Parani-ESD200, Sena, Seoul, Korea). The MCU controlled the ADC, which digitized all the signals in 16 bits at a sampling rate of 128 Hz and controlled the Bluetooth module to transmit the digitized data to a viewer application running on a tablet personal computer. The cost of implementing this mainboard was roughly EUR 200.

Each electrode of the two cECG probes (Figure 2d) included a conductive area (30 × 20 mm^2^) for the PCB and a high input impedance amplifier (OPA124, Texas Instruments, Dallas, TX, USA) with an aluminum cover, as shown in Figure 2d, to decrease ambient noise, as validated in the previous study [14]. The differential signal between a pair of electrodes was transmitted through insulated wires to a circuit to enhance the analog signal quality within the aluminum case (44 × 40 × 13 mm^2^). The signal was amplified by an instrumentation amplifier (INA118, Texas Instruments, Dallas, TX, USA) and filtered by a 4th Sallen–Key bandpass filter (i.e., 5–35 Hz) with operational amplifiers (OP497, Analog Devices, Wilmington, MA, USA) in the circuit. The cECG electrode pairs were placed on the backrest so that they would be in contact with the participant’s back area, and a seatbelt was used to make contact with the participant’s chest (i.e., halfway between the xiphoid and the umbilicus). Electroconductive fabric (27 × 16 cm^2^) was chosen as the common ground. The cECG system costs roughly EUR 200.

For each cPPG probe (Figure 2e), eight 850 nm LEDs (TSHG5210, Vishay, Malvern, PA, USA) were positioned around a PD (ODA-6WB-500M, Optodiode, Camarillo, CA, USA). The typical LED intensity was controlled by an 8 ch LED driver (TLC59210, Texas Instruments, Dallas, TX, USA), which supplied 100 mA for each LED with a typical forward voltage. Two cPPG probes were embedded in the seat base so that one probe (56 × 44 × 15 mm^3^) was in contact with the right hip area and the other was in contact with the right thigh area. The approximate cost of making the cPPG system was EUR 300. 

The BCG signal from the EMFi sensor (30 × 30 cm^2^) in Figure 2f was enhanced by a circuit of operational amplifiers (OP497, Analog Devices) for bandpass filtering and voltage gains. The circuit was composed of a 4th Sallen–Key highpass filter, a noninverting gain, and a 6th Sallen–Key lowpass filter. This filter circuit has been validated in several sleep studies using a polyvinylidenefluoride sensor for BCG measurements [10,15]. The price of the BCG sensor was about EUR 100. 

The total power consumption of the wheelchair system is approximately 1.5 W (i.e., 200 mW for cECG, 900 mW for cPPG, and 400 mW for the mainboard including BCG).

### 2.2. Reference Signals

In addition to the nonintrusive biosignals, the biochair system simultaneously recorded the reference signals—conventional ECG and PPG—using the same ADC. The conventional ECG was measured using attached Lead II Ag/AgCl electrodes (2223H, 3M, St. Paul, MN, USA) on both wrists of the participants. The reference PPG (PPG100C, BIOPAC) was measured using a PPG sensor strap (TSD200, BIOPAC) on the right index finger. To measure movement, the 3-axis acceleration values that occurred during motion tasks were measured by an inertial measurement unit (IMU) (EBIMU-9DOFV3, E2box, Hanam-si, Gyeonggi-do, Korea) affixed to the moving body part.

### 2.3. Digital Signal Process

**Figure 3:**
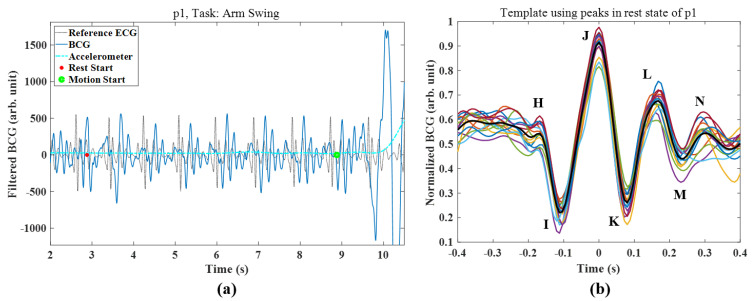
(**a**) BCG with reference ECG and accelerometer signals; (**b**) BCG template (bold) using serially validated peaks in the rest state of p1.

We processed and analyzed the dataset using MATLAB R2020b technical computing software (MathWorks, Natick, MA, USA). Bandpass filtering (5th order Butterworth) was applied to attenuate noise. The filters were used for cECG (8–13 Hz), seat cPPG (0.3–2.4 Hz), and BCG (0.6–5 Hz) to emphasize the systolic peaks, as shown in Figure 3.

The first 20 s of data should be excluded from all filtered signals to eliminate transitional waveforms; therefore, we started recording data 20 s in advance of the start time of the experiments. To distinguish task periods based on quantitative inspections, the mean absolute acceleration for the 3-axis acceleration data was calculated to avoid the directionality differences depending on each motion after lowpass filtering (1 Hz).

## 3. Methods

### 3.1. Template for Signal Quality Index

Using the system, we quantified the signal quality as an index. Methods of calculating the quality index of biological signals can be diverse depending on the target information. For example, the cross-correlation coefficient (*CC*) between a measured signal and a reference signal forms a clear method if we want to estimate bit-by-bit similarity to the conventional method [7].

However, we needed to consider the acquired signal characteristics through our unobtrusive methods without direct skin contact. Fundamentally, the material between the skin and the sensors causes signal morphology distortion even under ideal transmission conditions. This means that the signal’s morphological similarity with the reference could be less informative if we were to focus on timing accuracy (e.g., HR) of the specific peak occurrence during heartbeat cycles. Instead, we inspected how the signal reliability was affected by various motions using quantitative analyses of systolic peak detection results because the physiologically true peak is important for obtaining reliable heartbeat information. Additionally, biological signals have complex time-domain structures, which causes the separation of the signal and noise bandwidths in the frequency domain to be difficult in the signal-to-noise ratio (SNR) calculation. To overcome this ambiguity, a method to reconstruct ideal signals using the signal template and phase information from the reference ECG for SNR was introduced in [16] in an unconstrained measurement study. The templates were typically derived by averaging the systolic peak-to-peak signals by referring to the clear conventional signal with direct skin contacts. However, the goal of this research was to develop a convenient device that could assess the acquired signal reliability in everyday situations. For practical evaluations of signal reliability without a reference signal, we created a mixed method that implies the template and cross-correlation approaches.

When using the target signal only, verification of the systolic peak detections that define a heartbeat cycle was essential during template creation. For the validation procedure, instead of a reference, we applied the cross-correlation between peaks as detected by the Pan–Tompkins (PT) algorithm [17], which has been a basic detection method that is objectively reproducible within a specific time window. The PT algorithm determines a local peak as a systolic peak using adaptive thresholds (e.g., noise levels) calculated from early samples. If we choose a specific time span around a detected peak to inspect the signal shape, the signal candidates for the template can be defined for each peak.

### 3.2. Systolic Peak Comparison for Template Creation

**Figure 4:**
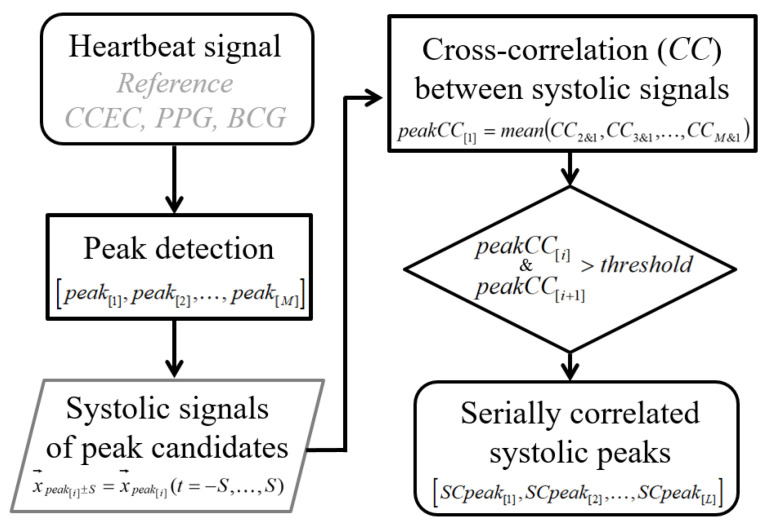
Preprocess to search serially correlated peaks.

Figure 4 shows a flowchart for comparing peaks using interpeak *CC*. The detailed procedures with equations are as follows.

Therefore, the similarity of the observed peak signal to the other peak signals represented as the mean *CC* for all peak combinations could function as the quality criteria when selecting a component to create the template.

*Cross-Correlation Between Signals Around Peaks.*

For a 6 s time window, the peak points were detected in their original order *i* = 1, …, *M* as

(1) $$\left[peak_{\left[1\right]},peak_{\left[2\right]},\ldots ,peak_{\left[M\right]}\right]$$

We stored *N* samples of the time series around each peak in a signal vector,

(2) $${\vec{x}}_{peak_{\left[i\right]}}=\left[x_{1},\ldots ,x_{N}\right]$$

The samples within the time span (*S*) around the peak are represented by

(3) $${\vec{x}}_{peak_{\left[i\right]}\pm S}={\vec{x}}_{peak_{\left[i\right]}}\left(t=-S,\ldots ,S\right)$$

The *CCs* were calculated using pairs of ${\vec{x}}_{peak_{\left[order\right]}\pm S}$ (i.e., two different *peaks*). The choice of *S* affects the observed signal morphology; here, it and was selected to be 0.4 s. For a given instance, the *CC* between the *peak*_[1]_ signal and the *peak*_[2]_ signal was denoted as

(4) $$CC_{2\&1}=corrcoef\left({\vec{x}}_{peak_{\left[2\right]}\pm S},{\vec{x}}_{peak_{\left[1\right]}\pm S}\right)$$

The M-1 number of *CC*s between each combination of two stored signals except the combination of itself was stored in a column (i.e., for each peak order). It yielded (5) of an (*M* − 1) × *M* matrix for *M* peaks.

(5) $$\left[\begin{array}{ccc} CC_{2\&1} & CC_{1\&2} & CC_{1\&M}\\ CC_{3\&1} & CC_{3\&2} & CC_{2\&M}\\ \vdots & \vdots & \vdots \\ CC_{M\&1} & CC_{M\&2} & CC_{M-1\&M} \end{array}\right]$$

*Mean Cross-correlation for Each Peak.*

We defined $peakCC_{\left[i\right]}$ as the mean value of the *CC*s in each column resulting from combinations, including ${\vec{x}}_{peak_{\left[i\right]}\pm S}$. For example, with $peak_{\left[1\right]}$, the average was calculated for all the *CCs* derived from combinations of ${\vec{x}}_{peak_{\left[1\right]}\pm S}\;$ and the other peak-centered signals in the first column of (5) for $\;peak_{\left[1\right]}$ as

(6) $$peakCC_{\left[1\right]}=mean\left(CC_{2\&1},CC_{3\&1},\ldots ,CC_{M\&1}\right).$$

Then, each $\;PeakCC_{\left[i\right]}\;$ was stored in each column.

(7) $$\left[peakCC_{\left[1\right]},peakCC_{\left[2\right]},\ldots ,peakCC_{\left[M\right]}\right]$$

*Template Formation Using Serially Correlated Peaks.*

The PeakCC  of the array in (7) indicated the stability of each systolic peak signal. To create reliable templates and HRs, the procedure must be conducted in continuously stable periods. Therefore, we defined serially correlated peaks ($SCpeak_{\left[j\right]}$) if both $\;peakCC_{\left[i\right]}$ and $peakCC_{\left[i+1\right]}$ exceeded a defined threshold (e.g., 0.6 for reference ECG) in the new sequence *j* = 1, …, *L* as

(8) $$\left[SCpeak_{\left[1\right]},SCpeak_{\left[2\right]},\ldots ,SCpeak_{\left[L\right]}\right]$$

The template was constructed using the average of these serially verified peak signals as follows:

(9) $$Template\vec{y}=mean\left({\vec{x}}_{SCpeak_{\left[1\right]}\pm S},\ldots ,{\vec{x}}_{SCpeak_{\left[L\right]}\pm S}\right)$$

Using the created template as shown in Figure 3b, we applied the template matching method to assess the signal quality.

### 3.3. Template Matching for the Signal Quality Index

Using the verified signal template, we then observed the similarity of the full data in the observation window to the template using template matching.

Consider a time series $\vec{z}$, whose sample index, *n* = 1,…,*N*, can be represented as

(10) $$\vec{z}=\left[z_{1},\ldots ,z_{N}\right]$$

The signal starting from index *n* in the observation period and with the same length as the template can be represented as follows:

(11) $${\vec{z}}_{\left[n\right]}=\vec{z}\left(t_{\left[n\right]}=0,\ldots ,2S\right)$$

**Figure 5:**
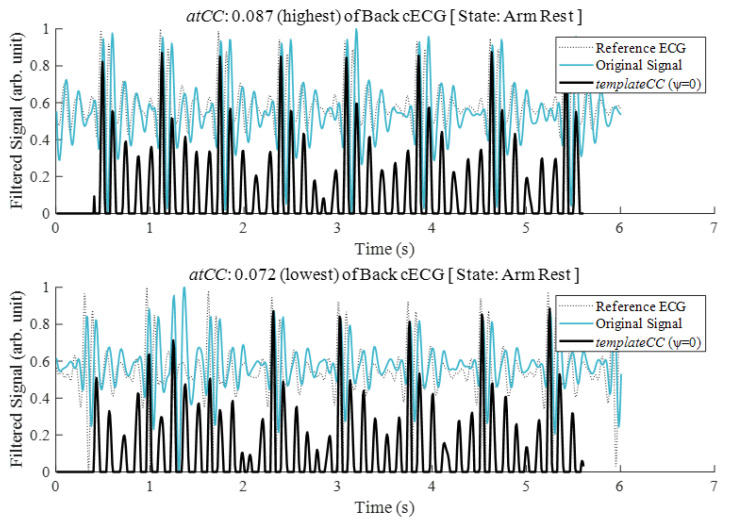
The highest (**upper figure**) and lowest (**lower figure**) values of the averaged template*CC* (*atCC*) derived from the original back cECG for resting states after completion of a motion task by a participant (p7).

The matching method yielded a new signal composed of the *CC* between the template and the observation period of the motion signal and included every other sample. We defined the *templateCC* (*tCC*) array as these *CC* results after template matching by substituting *templateCC* [*n*]= 0, where *CC* < ψ (i.e., a noise threshold, which is 0 for yielding the *tCC*, as shown in Figure 5) to eliminate noise:

(12) $$templateCC\left[n\right]=corrcoef\left(\vec{y},{\vec{z}}_{\left[n\right]}\right)$$

Then, the metrics for representing the *CC* between the template and the entire observed signal are

(13) atCC=Average(templateCC)

The quality decrease induced by the noisy state can be represented as the relatively noise-free state (i.e., upper figure of Figure 5) and the observation state:

(14) $$atCC_{diff}=\left|atCC_{noise\text{-}free}-atCC_{observ}\right|$$

Finally, $SQI_{tCC}$ was defined by the similarity between the averaged *tCC* in the noise-free signal and the observation signal window to consider the physiological characteristics:

(15) $$SQI_{atCC}=\frac{atCC_{noise\text{-}free}}{atCC_{noise\text{-}free}+atCC_{diff}}\cdot 100\;[\%]$$

In this paper, *SQI* denotes *SQI_atcc_.* By inspecting the signal quality, our goal was to enhance the possibility of achieving medical interpretation from reasonably qualified signals. Among the possible health information, an achievable target was to obtain reliable intervals between systolic peaks in heartbeats using the chair. In the present paper, *SQI* denotes $SQI_{atCC}$.

### 3.4. Utilization of the Templates in Systolic Peak Detection

When using the previous procedures to create templates, the PT algorithm was able to detect systolic peaks. However, we observed morphological differences between the measured signals and the conventional ECG. Unlike the ECG or PPG with direct skin contacts, the cECG and BCG signals were affected by media, which also caused distortions. In particular, the BCG measured by the EMFi sensor included a more confusing pressure signal composed of H-I-J-K-L-M-N waves. For example, the PT algorithm falsely detected peaks from the original BCG (Figure 3a) of a 6 s time window after the arm swing task of p1. This result occurs because the algorithm was designed to detect the outstanding peaks (i.e., R-peak) of clean conventional ECGs despite the removal function of the following less complex signal (e.g., T-waves) compared to L-M-N waves in BCG.

To solve this problem, we utilized the template matching method [18]. Figure 3b shows a template created from the average stable signals in a resting state. The *templateCC* yielded by the template matching is shown in Figure 5. The decreased complexity of *templateCC* compared to the original signal was the major effect of applying the method. This simplification occurred because the template was created with samples that centered the systolic peak among complex local peaks. Thus, the neighboring peaks of the original signal could disappear around the systolic peak in *templateCC*. Under the enhanced dominance of the systolic peak, *templateCC* was more appropriate for threshold-based peak detection than was the original signal.

### 3.5. Evaluation of HR Estimated by Detected Peaks

Physiologically, the timing of systolic peaks caused by physical blood transmission (i.e., PPG) and forces (i.e., BCG) were different from the electrical peaks of the reference ECG [7]. Therefore, using the timing similarity between each “single peak” estimated by dissimilar modalities is not recommended for making judgments of the success or failure of actual peak detections. Because of the above relationship, we evaluated the signal reliability as the “accuracy of HR” using estimated intervals between the detected peaks in each window. We defined the HR error (*HR_err_*) metric as the absolute difference between *HR_ref_* and *HR_est_*.

From the serially verified peaks ($SCpeak_{\left[j\right]}$), we calculated HR values in a time window. To show the distributions of the two simultaneous measurement results, we constructed a linear regression plot and a Bland–Altman (BA) plot of the reference HR (*HR_ref_*) and estimated HR (*HR_est_*) [19]. The Bland–Altman plot included horizontal lines showing the 95% limits of agreement for comparisons (the average difference is ±1.96 standard deviations of the difference). Specifically, the plot was illustrated using the Cartesian coordinates (BA(x,y)) of the derived values (i.e., the arithmetical mean and subtraction) using *HR_ref_* and *HR_est_*.

### 3.6. Experimental Condition and Movement Tasks

In total, 27 healthy participants volunteered for the measurement experiments with the biochair system. Participants were asked to sit on the chair, to fasten the seatbelt (Figure 2c), and to refrain from any motion in the resting state, including speech.

We asked participants to wear the clothes they would wear in their daily lives except for the reflective trousers, which were used to saturate the cPPG signal of the photodetectors. All the participants wore shirts or T-shirts over underwear in consideration of cECG compatibility [20].

Ten men (24–33 years old) participated in the quantitative movement experiments. The first movement experiment involved swinging (>30 cm/s) both arms to assess the effect of extreme upper-body motions during driving or while handling objects. The motion experiment was composed of 15 repetitions of alternating motion periods (6 s) and rest periods (6 s). The second movement experiment involved actual driving sessions at three speeds (i.e., 2 km/h, 4 km/h, and 6 km/h) in a powered wheelchair for a distance of 100 m inside a building. The 15 windows of resting data before the movement experiments were used for template creation. The outcomes of only nine participants were averaged to inspect the HR error because detachment of the reference ECG sensor was discovered to have occurred for one participant based on the measured signal. All the participants wore shirts or T-shirts in consideration of cECG compatibility [20].

Seventeen participants (ranging from 22 to 45 years old) took part in the virtual driving experiments. Virtual driving at a fixed speed (i.e., 95 km/h) along a straight path was adopted as the fundamental task. The drive continued for 55–70 min with no other cars or pedestrians present. The driving simulation software (Carnetsoft B.V., Groningen, The Netherlands) displayed the in-vehicle view. Participants were asked to keep the vehicle within its lane to test their reactions to the forced move at random intervals (5–19 s). Participants of the driving simulations wore prepared clothes made of cotton to minimize discomfort.

None of the participants had any psychological, neurological, or cardiopulmonary disorders. All the participants provided informed consent for inclusion before participating in the study. The study was conducted in accordance with the Declaration of Helsinki, and the study protocol was approved by the Institutional Review Board of the Seoul National University Hospital (IRB No. H-1509-117-705, C-1509-074-704).

## 4. Results and Discussion

### 4.1. Signal Quality Comparison

**Figure 6:**
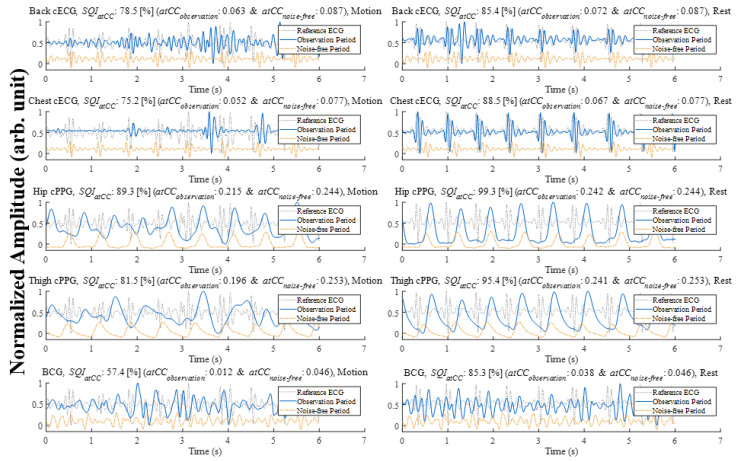
The signal quality index derived by the similarity of the average tCC of the original signals in each movement period (**left column**) and in the rest state after the arm motion task (**right column**).

We confirmed that the biosignal chair system successfully measured cECG, cPPG, and BCG during the initial noise-free state of all 23 participants. In the resting state, the shirts of all participants without outer clothing were compatible with cECG. Additionally, the cPPG apparatus showed the ability to obtain hemodynamic information from thigh vessels inside the trousers of all participants. However, the movement sessions yielded unstable physiological signals. For example, Figure 6 shows representative 6 s signals measured by an unobtrusive biosignal chair during arm movement as one of the extreme movements in the left column. The signal qualities of the back and chest cECGs and BCG were too noisy to detect systolic peaks as the *SQI* shows in Table 1. The *SQI* was able to successfully distinguish the difference between noisy states and noise-free signals. The *atCC* gave us information concerning signal measurement reliability because a lower *CC* represented a higher probability of noise interference or unstable contacts. Interestingly, in several periods, the cPPGs measured under the lower body were less distorted than were the other signals despite the upper-body motions. Unlike the BCG, which showed complex small differences between the systolic peak and the other peaks, the PPG proved to be far more robust under upper body part movements, resulting in *SQI* values over 80%.

Nevertheless, the right column of Figure 6 shows much higher signal stability and outstanding systolic peaks despite the fact that the signal was measured after the sensor detachments caused by motion. This result means that the unconstrained signal measured by the biosignal chair system in the resting time immediately after daily life motions has a satisfactory quality for interpreting medical status.

For the quantitative analysis, Table 1 shows the mean (SD) *SQI* for all the participants. On observation, the SQI of the hip cPPG averaged 11% higher than that of the thigh PPG during the arm motion state. One of the reasons for this result could be the higher contact stability of the hip as the location of the center of weight while sitting in a chair due to the proximity of the body part to the core of the body.

In the real driving of a powered wheelchair, a speed influence on *SQI* was expected. At the lowest speed (2 km/h), dominant systolic peaks were observed in cECG and cPPG. Both cECG signals showed an *SQI* of approximately 74%, and both cPPG signals yielded an *SQI* of approximately 85% for the 2 km/h driving task. A decrease in the *SQI* as a result of faster movement was observed except for the chest cECG with the seatbelt. Movements at higher speeds cause larger vibrations because the collisions between the faster wheel rotations and the corridor ground are affected by gravity. The decrease of *SQI* was a common phenomenon in all the signals except for the cECG on the chest because the belt maintained the pressure between the sensors and one’s chest. Because a seatbelt is essential for driving safely, the cECG on the chest could be a robust method under the less adequate conditions caused by high-speed movements.

The noise level in the BCG also reflected the greater impacts. Similar to real driving results, vocal movement caused by speaking affected the BCG because of the added vibration. Except for the BCG, all the other physiological signals during vocal movements achieved high *SQI* values—similar to the *SQI* values in the initial noise-free state and in the resting states after the motion states. This means that the biosignal chair system is capable of acquiring suitable cECG and cPPG signals during conversations, which occur frequently.

In the virtual driving task, the steering motions made during the lane-keeping task caused distortions of the cECG and BCG. The mean (SD) *SQI* of the cECG was 66.7% on the back and 79% on the chest. The *SQI* values were in the middle of the range of the extreme arm motion and resting tasks. Like real driving, the BCG was also distorted by the sensitivity of the EMFi sensor by the steering motions during the lane-keeping task.

We note that the *SQI* variations were not synchronized for all the types of sensors. As mentioned in the signal morphology during the upper-body movement experiment, cPPG has a strong point that compensates cPPG and BCG considering the diverse motions that occur during long-term monitoring.

For practical uses, cECG was inspected using each type of clothing (e.g., cotton, wool, and acrylic of 0.55–0.65 mm thickness) to frequency responses [4]. The noise level depends on clothing properties (i.e., impedance of the cloth). The impedance of the cotton shirt was less (with higher signal quality) than that of the acrylic shirt. In addition, cPPG were observed using various clothes that included cotton pants, blue jeans, and lower business suit (70% woolen and 30% rayon) in the range of 0.28 to 1.13 mm thickness [6]. In preliminary studies of the present system, thicker clothes lowered the signal quality of cECG and cPPG. This is because of the difficulty of transmitting electrons for cECG or photons for cPPG through thicker clothing. On the contrary, BCG which requires only physical pressure was less sensitive to the type or thickness of clothing.

### 4.2. HR Estimation with Screening Effects

For the systolic peak detection of unconstrained signals, the template-based method was applied with the threshold ψ to assess the signal characteristics. As mentioned in the Methods section, the threshold used for systolic peak detection affects the *HR_err_* and its coverage satisfies the criteria because the threshold could change the observed target *CC* area. The HR estimation results obtained by varying the threshold were analyzed, as shown in Table 2 and Table 3. Among the various signals, the cECG detected on the back and BCG were notably affected by the peak detection method during the resting states after arm movements. The *HR_err_* of the whole dataset and the absolute coverage gave us insights into the higher accuracy of *tCC* methods with a threshold. In this case, ψ = 0.3 was optimal for the back cECG and ψ = 0.0 was optimal for the BCG. False detection results by the conventional PT algorithm occurred because of the complex structure of the BCG morphology. The performance of template-based peak detection underwent a dramatic enhancement (i.e., ~20 BPM *HR_err_* decrease) in terms of the HR estimation accuracy. The absolute coverage experienced a 40% increase from the result of the PT method. Similarly, all sensors showed improved results of the mean *HR_err_* and coverage percentage with our methods.

Across all the biosignals measured during the virtual driving task, the absolute coverage and relative coverage that satisfied *HR_err_* < 3 BPM, according to the *SQI* criteria, are listed in Table 4. For example, the satisfactory window coverage among the absolute 6000 windows yielded by the BCG data was only 5.3% after screening by *SQI* > 90%. However, the satisfactory window coverage was 62.5% among relative windows after screening by *SQI* > 90%.

Almost all sensors showed higher relative coverage under the higher *SQI* screening except the chest cECG sensor. Hence, *SQI* inspection could cause a higher possibility of accurate *HR* estimations. However, too high of an *SQI* screening resulted in too small of an absolute coverage of sensors, such as 6.7% for the back cECG sensor. The driving pose taken to focus frequently caused detachments of the cECG sensor from the back, yielding an absolute coverage of approximately 22.2% after screening by *SQI >* 70%.

During the simulation task, the cPPG sensor achieved the highest coverage rate of 6 s windows among the physiological signals. This result occurred because the driving simulation required only steering motions without pedaling motions. Therefore, this cPPG result was not related to the dynamic driving; instead, it was related to the stable state or the upper-body movements that occur in daily life. Given the stable contacts in the cPPG sensors, both unconstrained cPPG sensors yielded an absolute coverage >65% of the total windows with the *SQI* > 80% screening criterion. The hip cPPG sensor without the fixation process achieved the highest relative coverage of 81% and absolute coverage of 43.0%. Similar to the *SQI* values in Table 1, the *HR*s resulting from the BCG were not reliable under sudden motions, producing an absolute coverage of only 5.3%. Thanks to the increase of *SQI* criteria from 70% to 90%, the relative coverage improved from 46.6% to 62.5%. This result is meaningful because the complex BCG morphology frequently confused the peak detectors and the estimated *HR* could not be informative without the proper screening process. After appropriate data curations, more accurate *HR* variability in the relative coverage can be used to detect the drowsiness using an artificially intelligent model in the previous study that yields the accuracy metrics of 98% [1].

### 4.3. HR Comparisons with Plots

**Figure 7:**
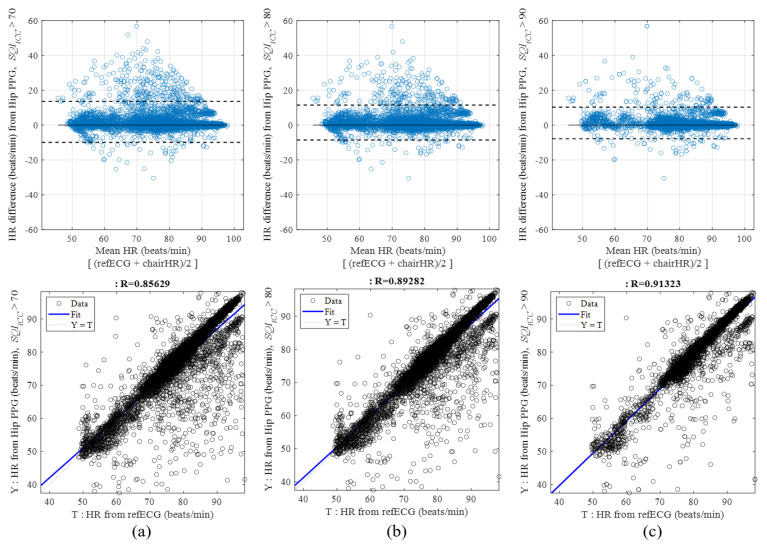
Bland–Altman and linear regression plots of HRs from the reference ECG signal and from the cPPG signals detected at the hip after screening by each *SQI_atCC_* criterion: (**a**) *SQI_atCC_* > 60%; (**b**) *SQI_atCC_* > 70%; (**c**) *SQI_atCC_* > 80%.

To compare the estimated *HR* by PT and the *tCC*-based peak detection methods with the reference *HR*, we analyzed the following plots (Figure 7). The *HR*s of 6000 hip cPPG windows (i.e., approximately 353 windows per participant) in the virtual driving session are illustrated as scatter plots. The bulk of the *HR* values were distributed along the ideal dotted line of the estimated *HR* (Y) of the hip cPPG sensor, equaling the target *HR* (T) of the reference ECG. The cross-correlation coefficient derived by the reference *HR*s and estimated *HR*s exceeded 0.91 which means that the estimated HRs were accurate to the reference HR.

The Bland–Altman plot shows wide data distributions because of the failure of systolic peak detection. The unusual *HR* errors occurred because the system did not always have stable sensor contacts even though the chest cECG sensor showed similar results to the cPPG sensors under upper-body motions. Through the inspection of the *HR* distributions from the leftmost (*SQI* > 70%) to the rightmost (*SQI* > 90%) figure, the elimination of the unusual *HR* errors by the higher *SQI* criteria was observed. The plot comparisons revealed that the *HR* estimations were more accurate after *SQI* screening of the unobtrusively measured signals. However, limitations of the *SQI* based on the *atCC* exist because it used the averaged template. The basic template could lose the individual traits. Hence, screening with the generalized aspect only unintentionally results in the deletion of the normal data that we want to use as an input for intelligence systems. Therefore, specific characteristics of the measurement modality must be considered [16,21,22]. We could expect that the procedure of adjusting optimal ψ thresholds implies individual traits.

## 5. Conclusions

To enable comfortable physiological signal monitoring, we implemented a biosignal chair that included multiple form factors. To quantitatively inspect the signal quality affected by the movement experiments, we created a similarity index to the stable signals based on a template constructed from serially correlated peaks. Additionally, using the *tCC*-based peak detection method to assess physiological information increased the HR estimation accuracy by utilizing the verified windows with an *SQI* criterion.

We investigated the reliability of signals affected by various motions using quantitative analyses of the systolic peak detection results to assess practical use situations. Our improved cPPG supplements the weakness of cECG and BCG. Through the experiments, we found the needs of multimodal sensors and the needs of optimal sensor selection algorithm in a further study.

In terms of compatibility, the results of the current study were limited to stable laboratory conditions. We controlled the temperature, humidity, electrical noise, and the clothing worn by participants to concentrate on the effects of movement in the experiments. Sensing conditions in real-life might include different motion types and varying levels of temperature, humidity, and electrical noise.

Despite the controlled conditions, we found the feasibility of the proposed signal quality index based on template CC for acquiring reliable information. The purpose of CC-based screening was the elimination of noisy signals. Therefore, CC-based screening is valid for the user group who have heart-related diseases (e.g., arrhythmia). With *SQI* screening, we could not delete windows that imply irregular systolic peaks but the windows that imply huge artifacts or disconnection.

The simple and general index could be utilized for all heart-related signals. Among the signals, the cPPG under various aspects of experimental conditions were relatively stable through inspections of *SQI* and estimated HR. This means the zero-effort measurement system could complement motion artifacts observed in signals of wearable devices.

## Figures and Tables

**Table 1:** $SQI_{atCC}\;$ of unobtrusive biosignals depending on each task.

(%) Biosignals	Initial Nose-Free	Arm Motion	Arm Rest	2 km/h Drive	4 km/h Drive	6 km/h Drive	Virtual Drive
cECG (Back)	94.4	68.9	84.4	74.1	73.4	71.3	66.7
cECG (Chest)	94.2	69.4	85.1	74.0	74.6	72.7	79.0
cPPG (Hip)	95.5	91.1	92.5	86.4	78.4	70.1	75.3
cPPG (Thigh)	95.8	80.4	93.7	84.4	81.0	76.3	75.1
BCG	93.9	50.1	51.1	51.4	51.5	50.9	67.4

**Table 2:** cECG *HR_err_* and absolute coverage of various *HR_err_* criteria for 6 s windows measured on back for each peak detection method in the resting states after arm motion.

	cECG Back	Absolute Coverage of Time Windows (%)		
Peak Detection Method	*HR_err_* (BPM)	*HR_err_* <5 BPM	*HR_err_* <4 BPM	*HR_err_* <3 BPM
PT	10.98	78.78	77.24	73.28
*tCC* (ψ = 0.0)	0.96	94.07	93.32	91.11
*tCC* (ψ = 0.1)	0.96	94.07	93.32	91.11
*tCC* (ψ = 0.2)	1.06	94.07	93.32	89.62
*tCC* (ψ = 0.3)	0.90	95.54	94.81	92.59
*tCC* (ψ = 0.4)	0.93	94.81	94.08	91.84

**Table 3:** BCG *HR_err_* and absolute coverage of various *HR_err_* criteria for 6 s windows measured depending on each peak detection method in resting states after arm motion.

	BCG	Absolute Coverage of Time Windows (%)		
Peak Detection Method	*HR_err_* (BPM)	*HR_err_* <5 BPM	*HR_err_* <4 BPM	*HR_err_* <3 BPM
PT	23.40	41.18	37.47	34.51
*tCC* (ψ = 0.0)	3.00	80.00	78.52	74.81
*tCC* (ψ = 0.1)	3.02	79.27	78.52	74.07
*tCC* (ψ = 0.2)	3.03	77.04	76.30	72.60
*tCC* (ψ = 0.3)	4.05	73.32	71.84	68.89
*tCC* (ψ = 0.4)	11.63	46.67	46.67	46.67

**Table 4:** Relative and absolute coverage (%) of *HR_err_* < 3BPM depending on the *SQI* conditions during the virtual driving task.

Biosignal *HR_err_* < 3 BPM	*SQI_atCC_* > 70% Screening		*SQI_atCC_* > 80% Screening		*SQI_atCC_* > 90% Screening	
	Relative Coverage (%)	Absolute Coverage (%)	Relative Coverage (%)	Absolute Coverage (%)	Relative Coverage (%)	Absolute Coverage (%)
cECG (Back)	22.2	22.2	22.0	19.6	25.8	6.7
cECG (Chest)	75.2	74.9	76.2	70.0	74.4	20.9
cPPG (Hip)	77.4	77.2	79.3	76.7	81.7	43.0
cPPG (Thigh)	66.9	66.8	69.2	65.9	76.2	39.0
BCG	46.6	39.6	54.3	23.0	62.5	5.3
